# Understanding Acceptability of AI Triage Tools Amongst Underserved Populations: Lessons From the Early Phases of Co‐Production With Bangladeshi Communities in Birmingham, UK

**DOI:** 10.1111/hex.70523

**Published:** 2025-12-04

**Authors:** Ian Litchfield, Gayathri Delanerolle, Lorraine Harper, Sian Dunning

**Affiliations:** ^1^ Department of Applied Health Sciences University of Birmingham Birmingham UK; ^2^ Birmingham Health Partners Birmingham UK; ^3^ University Hospitals Birmingham NHS Foundation Trust Birmingham UK; ^4^ West Midlands Health Technology Innovation Accelerator Birmingham UK; ^5^ Medical Devices Testing and Evaluation Centre, UHB NFT Birmingham UK

**Keywords:** co‐production, digital health, health inequalities, primary care

## Abstract

**Background:**

Effective communication is central to safe, high‐quality primary care. For Bangladeshi communities in the United Kingdom (UK), linguistic barriers continue to impede access to timely and culturally sensitive healthcare. This study describes an early phase of the co‐production pathway of the tool seeking to understand the contextual acceptability of an AI‐enabled translation tool designed for general practice, with functionality to capture symptoms, clinical problems, across diverse Bangladeshi dialects and provide guidance on next steps.

**Methods:**

We conducted a series of semi‐structured interviews with a sample of Bangladeshi patients from South Birmingham, UK to understand their attitudes towards using the AmarDoctor translation tool. The data were analysed using a directed content analysis to populate Sekhon's theoretical framework of acceptability.

**Results:**

Seven participants, all native Bengali speakers, were recruited. AmarDoctor was viewed positively for supporting appointment booking, guiding appropriate next steps, and offering a safe, anonymous means of discussing sensitive concerns. Noted strengths were its ability to capture symptoms in multiple Bengali dialects and its ease of use by those with limited digital literacy. The most frequently shared concern centred on the potential for translation inaccuracies and the subsequent risks.

**Conclusions:**

Participants expressed optimism about the role of AmarDoctor and similar AI‐enabled translation tools in improving access to primary care. To gain wider acceptance, AmarDoctor must maximise the next steps of the co‐production process that includes staff and commissioners to ensure translation accuracy meets the needs of all users and that credible pathways for implementation at scale are developed.

**Patient or Public Contribution:**

Patients and the public have been involved from the beginning of the AmarDoctor initiative, contributing to the design and content of patient facing materials, and informing the content of our topic guide.

**Abbreviations**
AI: artificial intelligence
HICs: high income countries
NHS: National Health Service
TFA: theoretical framework of acceptability
UK: United Kingdom

## 1 Background

Artificial intelligence (AI) (which can be broadly defined as a computer system which can perform tasks usually requiring human intelligence) is increasingly shaping the delivery of clinical care worldwide [1, 2, 3, 4]. It is expected to improve health system performance and patient outcomes across a range of healthcare settings, domains and applications [5, 6]. In the United Kingdom (UK), the National Health Service (NHS) has embraced AI technologies as central to its digital transformation strategy and latest 10‐year plan [7, 8].

Amongst the most visible of these technologies are patient‐facing AI‐driven symptom checkers and virtual assistants [9, 10, 11]. These platforms use symptom input from patients to provide possible diagnoses and guide triage decisions with an increasing number being used in high‐income health systems [12, 13] and growing evidence of their benefit in primary care settings [9, 14, 15, 16]. In the NHS their potential is of particular interest to general practice, where rising demand, and workforce shortages frequently leave those patients seeking care enduring lengthy waits on busy telephone systems, being triaged by nonclinical ‘reception’ staff, or otherwise expected to navigate online booking systems regardless of their digital literacy [17]. However, the implementation of symptom checkers and virtual assistants is not without challenges, with concerns amongst patients around their accuracy, safety and transparency [16, 18, 19]. These issues are particularly pronounced amongst underserved populations, defined here as those who are economically deprived and/or from ethnic minorities that are engaged less effectively by formal healthcare interventions [20]. These populations also encounter more pronounced barriers to the access and utilisation of digital health innovations related to access of digital devices and infrastructure [21], compounded by their affordability [22], varying levels of confidence and sophistication in the use of digital devices [23] and their failure to accommodate specific linguistic, and cultural needs [24].

There are a number of ways that AI‐enabled digital health tools might successfully accommodate some of these challenges including accompanying their implementation with free or discounted data, hardware or targeted and tailored training [25]. However, to better understand and meet the cultural and social sensitivities of patients with diverse health literacies, and understanding of AI enabled care [26, 27], it has been recommended they are coproduced with their target populations [28, 29]. Evidence of the benefits of co‐production in digital health is growing [30, 31, 32], though few such co‐production approaches have specifically targeted underserved populations [33, 34, 35, 36, 37]. The work presented here describes valuable learning from the early stages of co‐production of a primary‐care‐based AI‐enabled translation and symptom‐checking platform, specifically designed for UK based Bangladeshi populations accessing primary care [38]. It uses qualitative data gathered from a series of semi‐structured interviews to understand patient attitudes, expectations and preferences of such an AI tool, the recognised first phase of co‐production [39]. It is analysed using a recognised theoretical framework of acceptability (TFA), and we place these finding in the context of existing evidence and conclude with recommendations for the next phases of co‐production of this and similar tools being developed elsewhere for similar minority populations.

## 2 Methods

### 2.1 Study Design

This qualitative study employs semi‐structured interviews to explore the experiences of the AmarDoctor tool used by a Bangladeshi population residing within Birmingham as the first stage of a co‐production exercise intended to develop and refine a primary care based chatbot targeting Bangladeshi communities in the United Kingdom. It used a directed content analysis to populate Sekhon's theoretical framework of acceptability [40, 41]. Ethical approval was granted from the Science Technology Engineering and Mathematics Ethics Committee, University of Birmingham (ERN_3508‐Dec2024).

### 2.2 Summary of the AmarDoctor Tool

**Figure 1:**
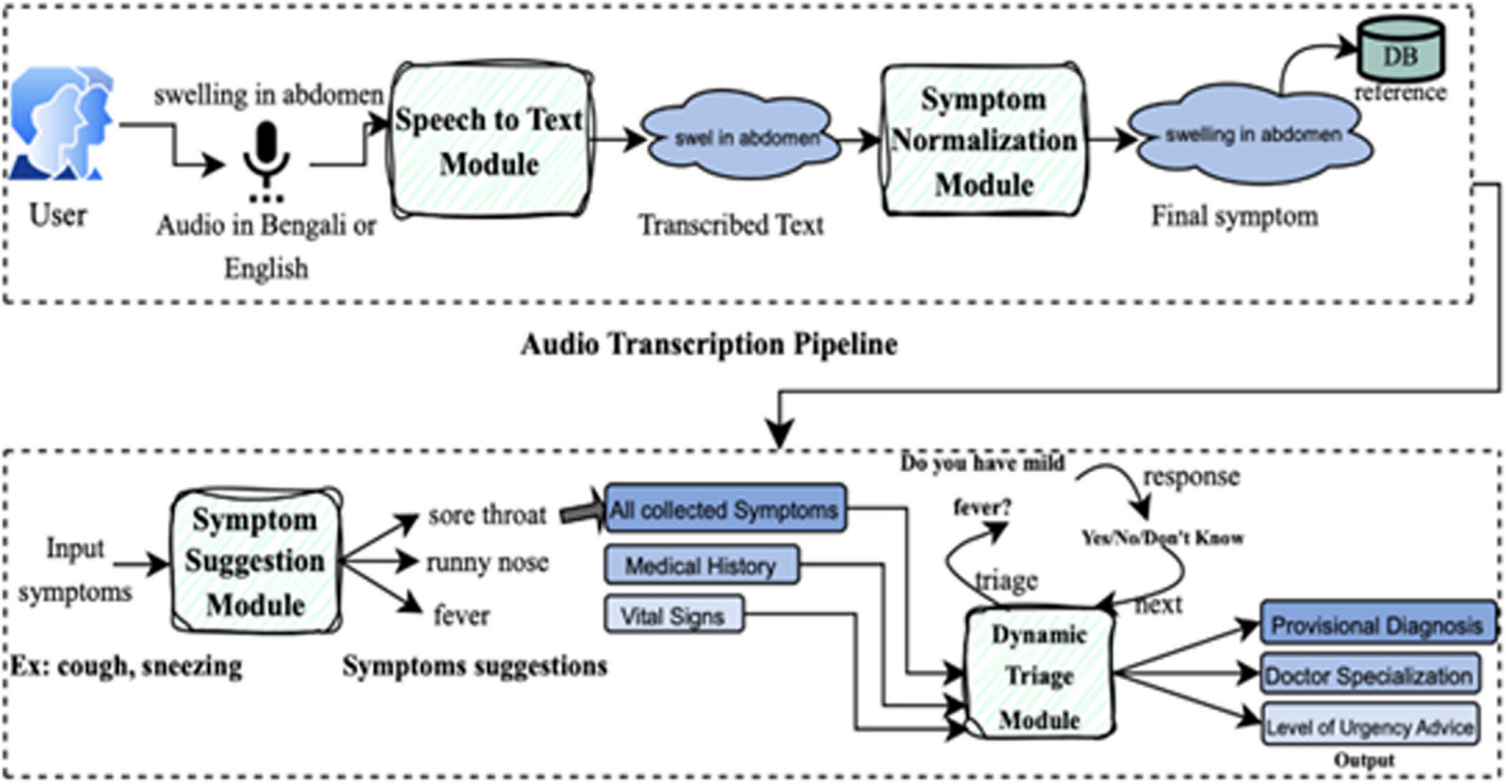
Outline of AmarDoctor tool.

The tool is a prototype, multilingual, AI‐enabled, voice‐assisted symptom‐gathering platform designed to support Bengali‐speaking communities in the United Kingdom, particularly Bangladeshi and other South Asian groups who face language barriers in primary care. It enables patients to report symptoms in either English or Bengali through text or voice input. The system then generates a translated summary of the patient's presenting complaint, which can be securely shared with general practice staff before consultation and otherwise facilitate referral to appropriate healthcare staff. At the core of the system is a deep learning‐based Bengali medical speech‐to‐text engine capable of recognising clinical terms in multiple dialects. The typical patient journey is shown in Figure 1, which illustrates the intended flow of symptom entry, translation and clinical decision support within general practice pathways.

### 2.3 Setting/Recruitment

The study took place in South Birmingham, a large and diverse city in the UK's West Midlands. The wards in the south of the city are amongst some of the most populous in the city, with high levels of deprivation and a superdiverse population with a large south Asian community [42]. Participants were convenience sampled from Bangladeshi communities using two key routes: The first was via a large local iftar event (recruitment was pursued during Ramadan) and the second was via local faith and community organisations such as the Bangladesh Islamic Centre and the Bangladesh Women's Association. All participants who expressed an interest either approached the second author (G.D.) directly at the Iftar event or after being provided with contact details via the community organisations. Those who were interested were provided with a participant information sheet and the opportunity to ask questions before supplying signed consent in line with the study's ethical permissions. During the process a number of barriers to participation were described by female participants this included the final decision on their involvement being made by senior male members of their family, which required reassurance of these individuals before consenting female participants. There were also issues relating to arranging interviews due to familial responsibilities with several unable to attend at short notice leading to interviews being conducted in the evenings.

### 2.4 Data Collection

Interviews were conducted by GD a researcher in health services trained and experienced in qualitative research methods who was unknown to participants before the study. Guided by the principles of data adequacy, the number of participants sought was informed by the narrow focus and aim of the work [43]. The intention was to conduct sufficient interviews to provide a data set that was representative of a range of ages, genders and digital experience sufficient for this early phase of co‐production [44, 45]. To understand exactly how well the sample represented a range of perspectives a number of demographic characteristics were collected on each participant alongside their consent, this included their age, gender and educational attainment. The interviews were conducted via telephone or via the Teams video platform using a topic guide informed by existing literature on digital health adoption and AI use in minority populations. Questions included existing experiences of accessing care, perceptions and utility of AI, their comfort and experience in speaking to a nonhuman interactive system, and the level of trust they might place in such a system (see Box 1). The interviews were digitally audio‐recorded, and transcribed verbatim and the data managed using Excel.

BOX 1 **Summary interview topic guide.**

1 *Questions relating to usual routes/barriers accessing primary care*

- *What is your experience in attempting to secure an appointment at your local general practice?*
- *How do you feel about the conversations you have with your GP practice staff?*
- *What if any is your experience of existing digital access systems such as online booking?*
*Questions on using an AI tool*
- *How do you feel about an AI tool supporting your communication with your GP or other practice staff?*
- *Do you feel comfortable speaking to a nonhuman interactive system?*
- *What are your preferences for how the tool can be utilised?*
- *What concerns if any do you have about the tool?*

### 2.5 Data Analysis

The data will be understood through TFA developed by Sekhon et al. to explore acceptability of health care interventions from the perspectives of providers and patients [41]. It consists of seven domains: Affective attitude, burden, ethicality, intervention coherence, opportunity costs, perceived effectiveness, and self‐efficacy. Together they describe whether those receiving (or delivering) an intervention consider it to be appropriate based on anticipated or experiential cognitive and emotional responses [41]. The data were analysed using a directed content analysis, populating the TFA using Elo and Kyngas's open matrix approach that allowed for the inclusion of emerging constructs within existing domains [40, 46]. The process was collaborative, with GD and the first author (I.L.) independently allocating data within the framework with the final allocation consensually agreed by all authors.

## 3 Results

### 3.1 Summary of Participants

A total of seven participants were interviewed with their lasting between 15 and 30 min. Participants consisted of four women and three men. Of the five respondents that provided demographic data their age ranged from 28 to 58 years, with all identifying as Bengali Muslims, residing in wards of 7th decile of Index of Multiple Deprivation, were employed either full‐time or part‐time, with a range of educational attainment including two with postgraduate qualifications. Alongside English, four spoke Sylheti and one Bangla. The characteristics of participants are summarised in Supporting Information 1.

### 3.2 Qualitative Data

Following best practice in adapting frameworks to specific contexts [47], the TFA was refined to bring greater clarity and concision: the domain ‘*Ethicality’* has been renamed *‘Cultural sensitivity’*, a phrase more precisely describing the domain and its constructs as represented in this study. Due to the overlap in the findings and their definition in the original framework, *‘Burden’* and ‘*Opportunity Costs’* were combined to form one domain, as were the similarly related domains of ‘*Affective attitude’* and ‘*Intervention coherence’*. No data were relevant and therefore allocated to the domain *‘Self‐efficacy’*. Therefore, including *‘Perceived effectiveness’* there was a total of four final domains containing 11 constructs. Table 1 presents definitions of the four domains, emergent constructs and a summary of the underpinning evidence. These domains are further described below with illustrative quotes describing each of their constructs with participants described by their ID, gender and age respectively.

**Table 1:** Summary of findings.

Domain	Definition	Emerging construct	Participant responses
1. Affective attitude and intervention coherence	How participants feel about (AI‐enabled) interventions and how far the intervention and its construct are understood	1.1 Familiarity with using digital resources	Familiarity of using search engines to seek information on health conditions Younger participants described their comfort in interacting with AI tools including synthesised voices
		1.2 Content of intervention	Functionality of the tool was widely understood
2. Burden/Opportunity costs	Perceived amount of effort required to use the intervention	2.1 Preferable to usual routes	Straightforward compared to using digital platforms More comfortable than talking to nonresponsive staff
		2.2 Reduced wait times	Precluded long waits on telephone, or for consultations
3. Cultural sensitivity	How well intervention aligns with individual beliefs and values	3.1 Attitudes to sharing health information	Happy to share confidential information as seen as non‐judgemental/confidential so
		3.2 Value of multi‐lingual interface	Better able to describe symptoms using primary language
4. Perceived effectiveness	How well the intervention can meet user needs and preferences	4.1 Facilitates accessible and timely access	Tool offers more timely and appropriate access for a range of south Asian communities
		4.2 Implications of inaccuracy	Whether the translations are accurate, or too concise Lack of opportunity to question its advice

#### 3.2.1 Affective Attitude and Intervention Coherence

##### 3.2.1.1 Familiarity With Using Digital Resources

In contemplating seeking health information and advice from nonclinical sources, younger participants described their familiarity with seeking a range of information from online search engines. This included health advice where they would frequently seek initial information on a particular symptom or health concern from online search engines. Younger respondents went on to describe their familiarity and comfort with using popular AI interfaces:

I have used ChatGPT… I found like they're quite useful … In terms of… help and rapid suggestion it is… it is great.

P01, M, 28 years

##### 3.2.1.2 Content of Intervention

Participants demonstrated clear understanding of the various aspects of the tool's purpose and functionality, including its role in translation, symptom description, appointment facilitation or referral. As one participant summarised:

If I tell this app my problem and it will pass my problem to doctor… it will set an appointment… much easier for me.

P04, Male, 29 years

#### 3.2.2 Burden/Opportunity Costs

##### 3.2.2.1 Preferable to Usual Routes

In considering the use of AmarDoctor, participants highlighted the frustrations they had in speaking to reception staff who before they could explain the issue would refer them to the online appointment booking system:

Whenever I call for appointment, they say go to [online booking] app and cut the call instantly.

P04, M, 29 years

For others, it was felt that the AmarDoctor tool offers a favourable alternative to speaking to staff that might not understand their concerns, or their language, or otherwise they felt they were being judged or not being taken seriously. As one participant explained:

I am very, very ill. I can't explain… everybody thinking … I am just a… attention seeking.

P06, Woman, (undisclosed age)

The challenges of communicating their symptoms or issues over the phone was echoed by another participant who felt communicating with a bilingual chatbot would be preferable than staff with whom they could not communicate in their native language:

[I can] tell this app my problem… it will be easier.

P05, Woman, 39 years

##### 3.2.2.2 Reduced Wait Times

Beyond the concerns of language or using digital interfaces, an issue shared by many participants were long waits on busy telephone lines and anxiety inducing system level delays when seeking a consultation with a clinician:

My son… vomiting… we had to wait for seven hours… that was like a nightmare for me.

P04, Male, 29 years

Experiences such as these meant that many participants felt the tool could provide more timely advice or care navigation, as one noted, it could ‘*…initially save my time… to [point me in the] right direction’.* P01, Male, 28 years

#### 3.2.3 Cultural Sensitivity

##### 3.2.3.1 Attitudes to Sharing Health Information

Participants understood that AI driven tools such as AmarDoctor can offer an opportunity to describe medical issues challenges and symptoms that might be considered difficult to discuss in their, and other South Asian cultures [48]. Our participants reported barriers to seeking help for mental health issues due to the stigma attached ‘*That tool could have saved time and my health*’ *(P03, Woman, 58 years)*. As another participant confirmed, the tool was described as a valuable and safe first point of contact, enabling users to express sensitive issues safely and anonymously.

At that moment, I didn't want to speak to anyone… If I had this tool, I would use it first before [speaking to] anyone else.

P01, 28,M

##### 3.2.3.2 Value of Multi‐Lingual Interface

AmarDoctor was valued for allowing participants to converse in their natural language and dialect. This was not because they were necessarily expected to speak in English but that the second languages used by providers were not reflective of their needs as Bangladeshis using other languages from South Asia:

One doctor… explained to my dad… in Hindi, Urdu… But they don't talk… they don't talk about our language.

PID2

In contrast, the tool was valued for presenting the opportunity to speak in their native language *‘They translate it… I speak Bengali then… they translate the English’.* P07, Male (age undisclosed)

#### 3.2.4 Perceived Effectiveness

##### 3.2.4.1 Facilitates Accessible and Timely Access

The AI tool was widely perceived as a means of more effectively facilitating triage, or access to reliable guidance. (‘*They don't know where to go? … it could refer [them]… which could save the time and health risk as well’.* P01, Male, 28 years). Another participant echoed the need for this tool across South Asian communities:

Most of the people… Bengali people or Indian people… need this program… [its] helpful to our communities.

P07, Male, (years undisclosed)

##### 3.2.4.2 Implications of Inaccuracy

Concerns were expressed around the implications of the tool mistranslating their symptoms or the reason for seeking an appointment and direct them to the wrong member of the practice team or otherwise fail to offer accurate advice. One participant was concerned that their dialectal description of a symptom might not be mistranslated into English:

[they might not] …understand the exact word… whether that really translates to English or not…If the translation is not clear, especially for medical words, it won't help the doctor understand me properly.

P02, Woman, 34 years

There were also concerns that even if accurate, the AmarDoctor translation would be too concise and lose valuable context in how it understood the symptoms. *‘Must be detailed, not too short’* (P03, Woman, 58 years). Ultimately, one participant wondered if the tool could respond to further questions where previously they would seek clarity or reassurance by questioning their General Practitioner:

I ask the doctor – “Please explain to me properly” ‐ …that's my main thing.

P02, Woman, 32 years

## 4 Discussion

### 4.1 General Findings

The AmarDoctor tool is novel in the context of many existing AI health applications currently being developed in the United Kingdom which rarely integrate community‐informed cultural design with a strong translation capacity for primary care triage. The TFA proved an effective and structured means of analysing the qualitative data gathered from this initial stage of co‐production. Participants described their broad familiarity with AI tools and the specific functionality of AmarDoctor (*Affective attitude and intervention coherence*); the ease and speed of its use in comparison with usual routes of access (*Burden/Opportunity costs*); favoured its objective confidentiality and ability to understand their primary language (*Cultural sensitivity*); and understood its ability to provide safe, effective and efficient access (*Perceived effectiveness*). Below we explore these findings in the context of existing information, describe policy implications of the approach and the next steps in its co‐production.

### 4.2 Specific Findings

#### 4.2.1 Affective Attitude/Intervention Coherence

Participants familiar with digital technologies were comfortable with the concept of AI and its use in health care, reflecting existing evidence of favourable patient perspectives on AI health tools [49] including their use in accessing care [50]. That the concept of the tool was welcomed by younger participants reflects broader patterns of higher uptake of digital health tools in younger patients [51, 52, 53]. It is likely that more work will be needed to raise awareness and reassure other sectors of the target population on the use of AmarDoctor and similar tools. This process might include understanding patient specific levels of digital literacy, ideally by using one of several tools available [54] increasing awareness of what digital health constitutes and entails [24, 51] and providing targeted training for elderly users [55].

#### 4.2.2 Burden/Opportunity Costs

Participants consistently described their preference for using the tool in the context of widely reported issues of difficulties reaching reception staff on busy telephone lines [56, 57]. Participants were also unhappy at being directed to online booking platforms and there is previous evidence that these platforms are underutilised and unsuitable amongst underserved populations, reflecting a broader ‘digital divide’ in the take up of health‐related digital tools [58, 59]. They also described the issues they had conversing with members of the staff that didn't speak their language, again this apathy toward ‘gatekeeping’ reception staff is widely reported in UK primary care, particularly amongst ethnic minorities [56, 60].

#### 4.2.3 Cultural Sensitivity

Many patients attempting to book a consultation over the phone are typically required to describe their symptoms to a receptionist and they have expressed concerns over relaying sensitive medical information to nonclinical members of staff [56, 61]. In contrast participants described how they appreciated the perceived anonymity and neutrality of a digital tool and its’ appearance of a safe space to talk more freely about sensitive medical issues such as their mental health. This is particularly valuable in South East Asian populations where there is heightened stigma attached to mental health issues [48], and where traditional solutions for bridging language barriers, such as interpreter services, or reliance on family members raise issues of confidentiality, and cultural acceptability [62].

Research has consistently linked lower satisfaction with health services with issues relating to language, communication and cultural understanding [63, 64]. Older Bangladeshi patients have been noted to give poor ratings to doctor‐patient communication, often due to language barriers [60]. In this context an AI‐driven consultation assistant that could be programmed in Bengali or Sylheti language was welcomed by participants as a means of allowing them to describe their symptoms more accurately by using the language more familiar to them. A factor recognised by the Royal College of GPs that recommends natural language processing which enables real‐time translation for improving access for non‐English‐speaking patients [65].

#### 4.2.4 Intervention Coherence/Perceived Effectiveness

Participants were overwhelmingly in favour of using the tool to access services, understanding as previous evidence has indicated with similar tools, that it offers a safe, effective and efficient alternative means of accessing primary care [9, 14, 15]. Effective communication is a cornerstone of high‐quality healthcare [66] and among Bangladeshi populations, in the United Kingdom, language is cited as a persistent barrier to accessing care [67, 68]. By addressing communication barriers and improving clinical interactions, the tool can contribute to more inclusive and culturally attuned services. Not only can it enhance access for Bangladeshi populations, but given the UK's richly diverse population, encompassing some 287 recognised ethnicities [69]. AmarDoctor also holds the potential to evolve into a multilingual translation platform, able to support equitable access [70]. However, the success and safety of AI powered symptom checkers and triage tools are predicated on accurate translation [71, 72]. Although many participants had prior experience with digital translation tools, trust in AI translation for medical consultations was conditional on the accuracy of it transcribing medical terminology and whether translations would capture the full intent and nuance of their conditions. There are legitimate concerns about algorithmic biases if AI systems are trained predominantly on data from other demographics [73] and ultimately such concerns will only overcome by coherent messaging informed by robust messaging [6].

### 4.3 Service Implications

From a policy perspective, this study presents both an opportunity and a challenge to the current UK AI in Healthcare Framework, which emphasises safety, transparency and performance metrics but does not adequately account for cultural competence and linguistic accessibility as core evaluation criteria [74]. Existing national strategies, such as NHS England's *Artificial Intelligence in Health and Care Award* and the *Topol Review* on digital readiness, prioritise scalability and clinical utility but offer limited guidance on equitable deployment in diverse populations [75]. This study underlines the benefits (and challenges) of embedding inclusivity and community‐derived design principles into AI health tools from inception. It is also possible that co‐production initiatives in high income countries (HICs) can learn from those conducted in low‐ and middle‐income settings amongst populations similar to those minoritised in HICs where its’ potential to develop equitable care is increasingly being recognised [76, 77]. These include examples in Africa [78, 79] and India [80, 81] and have begun to include co‐production specific to digital health [82, 83].

Using the TFA sets a precedent for redefining AI evaluation standards to consider acceptability as an equity‐based metric. For developers this suggests a shift from viewing cultural and linguistic adaptation as ancillary features to recognising them as central determinants of the design and implementation of AI in healthcare systems, aligning with the NHS aims of the *Core20PLUS5* approach to narrowing health disparities [84].

Specific to AmarDoctor, the next phase will focus on further testing the accuracy of the tool and co‐producing an implementation pathway. This will involve using best practice in community engagement to create a long‐standing dialogue with Bangladeshi communities in Birmingham [85]. They will in turn contribute to the next phases of co‐production to refine AmarDoctor's interface, ensuring that translations are clinically accurate and culturally appropriate. These ensuing phases will also include a range of primary care staff and commissioners to ensure IT infrastructure needs, the role and training of staff, the protocols for its use, and the financial model that will support its implementation at scale are understood and accommodated.

### 4.4 Strengths and Limitations

Themes emerging from participant interviews highlight key concerns and potential advantages related to access, communication, cultural alignment and digital familiarity of the AmarDoctor tool. Populating the TFA using an open matrix approach with two researchers coding independently bolstered analytical credibility and trustworthiness [40, 46]. The authors acknowledge that the number of participants were lower than anticipated and although this may be considered to limit broader generalisability, consensus theory tells us that a smaller number of ‘experts’ with shared knowledge of a tightly defined topic are needed in describing common experiences and values [86]. In the context of the co‐production of AmarDoctor this means the data are able to offer valuable insight transferable to similar populations and contexts [86, 29] without the collection of superfluous data [87, 88].

That we were able to reach the target community at all underscored the importance of working with trusted community leaders and adopting culturally sensitive approaches. The target Bangladeshi community in South Birmingham possessed strong internal networks and cultural cohesion, which create trusted spaces for interaction and information‐sharing about the project, but the same insularity made external recruitment efforts difficult. These were further impeded by cultural norms where participation was guided by male family members, reducing direct access to women and older adults and highlighted the challenges of co‐production with underserved communities [33, 34, 35, 36, 37]. There are a number of ways these challenges might be addressed when recruiting underserved populations to digital co‐production exercises [89]. These include understanding the additional time needed to raise awareness of digital health amongst those with little previous experience [90], the limits of resource and opportunity that can impact the ability of underserved populations to contribute (perhaps by offering support in terms of transport or childcare) [90, 91], the use of interpreters [92] and encouraging attendance by using flexible timing and location of sessions, and accessible venues [93].

## 5 Conclusion

This study reports the findings from a useful first step in the digital co‐production process, describing a layered acceptability of the AmarDoctor tool amongst participants and demonstrating the difficulty and value of engaging underserved groups in digital health co‐production. The next phase of the co‐production process will further develop the utility and usability of the tool using iterative co‐design workshops involving patients, carers, clinicians and interpreters. The challenges of recruitment we faced demonstrate the need for a more long‐standing dialogue with the Bangladeshi community in Birmingham, one that offers and delivers tangible benefits of their engagement in their health and care. This should use best practices in community engagement and involve regular and equitable working with primary care staff and commissioners to develop trust, shared learning, and ultimately the financial model to support its implementation at scale that is affordable for patients and commissioners.

## Author Contributions

**Ian Litchfield:** methodology, formal analysis, supervision, writing – original draft, writing – review and editing. **Gayathri Delanerolle:** conceptualisation, methodology, investigation, formal analysis, data curation, writing – review and editing, investigation. **Lorraine Harper:** supervision, funding acquisition, writing – review and editing. **Sian Dunning:** conceptualisation, supervision, funding acquisition, writing – review and editing.

## Ethics Statement

Ethical approval was granted from the University of Birmingham Science Technology Engineering and Mathematics Research Ethics Committee (ERN_3508‐Dec2024).

## Consent

Written informed consent was obtained from all participants including permission for their data to be used in accordance with that consent for the use of this publication.

## Conflicts of Interest

The authors declare no conflicts of interest.

## Supporting information

Supplementary File 1 Summary of participant characteristics v23.09.25.

## Data Availability

Data are available upon reasonable request from the corresponding author.
