# Supplementary material for: Understanding Acceptability of AI Triage Tools Amongst Underserved Populations: Lessons From the Early Phases of Co‐Production With Bangladeshi Communities in Birmingham, UK
Source: Health Expect. 2025 Dec 4;28(6):e70523. doi: 10.1111/hex.70523 (PMC12676503; doi:10.1111/hex.70523)
Supplement: Supplementary file 1 — Summary of participant characteristics v23.09.25. [file HEX-28-e70523-s001.docx]

**Supplementary File X Summary of participant characteristics**

| **Participant ID** | **1** | **2** | **3** | **4** | **5** |
| --- | --- | --- | --- | --- | --- |
| **Age** | 28 | 34 | 58 | 29 | 39 |
| **Gender** | Man | Woman | Woman | Man | Woman |
| **Ethnicity** | Bengali | Bengali | Bengali | Bengali | Bengali |
| **Religion** | Islam | Islam | Islam | Islam | Islam |
| **Index of Multiple Deprivation (decile)*** | **7** | **7** | **7** | **7** | **7** |
| **Relationship Status** | Single | Single | Married | Married | Married |
| **Family Status** | Other | Nuclear | Nuclear | Other | Nuclear |
| **Education** | Postgraduate | Undergraduate | Secondary education | Postgraduate | Undergraduate |
| **Employment Status** | full-time | part-time | part-time | full-time | part-time |
| **Sector** | Construction/ Academic | Hospitality | Hospitality | Accounts | Hospitality |
| **Bilingual**  **Status** | Native Sylheti | Native Sylheti | Native Sylheti | Native Bangla speaker | Native Sylheti |
| **Health Conditions** | No | No | Yes | No | No |
| **Carer** | No | No | Yes | No | No |
| **Care-giver** | No | No | Yes | No | No |
| **Disabilities** | No | No | Yes | No | No |

**Where 10 is most deprived*
